# Pharmacological Approaches in Neurofibromatosis Type 1-Associated Nervous System Tumors

**DOI:** 10.3390/cancers13153880

**Published:** 2021-08-01

**Authors:** Omar Rabab’h, Abeer Gharaibeh, Ali Al-Ramadan, Manar Ismail, Jawad Shah

**Affiliations:** 1 Insight Research Institute, Flint, MI 48507, USA; omar.rababh@iinn.com (O.R.); abeer.al-gharaibeh@iinn.com (A.G.); medsali@umich.edu (A.A.-R.); manar.ismail@iinn.com (M.I.); 2 Center for Cognition and Neuroethics, University of Michigan-Flint, Flint, MI 48502, USA; 3 Insight Institute of Neurosurgery & Neuroscience, Flint, MI 48507, USA; 4 Insight Surgical Hospital, Warren, MI 48091, USA; 5 Department of Medicine, College of Human Medicine, Michigan State University, East Lansing, MI 48824, USA

**Keywords:** neurofibromatosis type 1, neurofibroma, malignant peripheral nerve sheath tumor, glioma, tumor microenvironment, targeted therapy, noncoding RNA, gene therapy, NF1 models

## Abstract

**Simple Summary:**

Neurofibromatosis type 1 (NF1) is a common cancer predisposition genetic disease that is associated with significant morbidity and mortality. In this literature review, we discuss the major pathways in the nervous system that are affected by NF1, tumors that are associated with NF1, drugs that target these pathways, and genetic models of NF1. We also summarize the latest updates from clinical trials that are evaluating pharmacological agents to treat these tumors and discuss the efforts that are being made to cure the disease in the future

**Abstract:**

Neurofibromatosis type 1 is an autosomal dominant genetic disease and a common tumor predisposition syndrome that affects 1 in 3000 to 4000 patients in the USA. Although studies have been conducted to better understand and manage this disease, the underlying pathogenesis of neurofibromatosis type 1 has not been completely elucidated, and this disease is still associated with significant morbidity and mortality. Treatment options are limited to surgery with chemotherapy for tumors in cases of malignant transformation. In this review, we summarize the advances in the development of targeted pharmacological interventions for neurofibromatosis type 1 and related conditions.

## 1. Introduction

Neurofibromatosis type 1 (NF1) is a common autosomal dominant genetic disease with complete penetration. NF1 is associated with significant morbidity and mortality, and the clinical features of the disease vary from mild to severe [1,2,3,4,5,6]. NF1 is caused by a mutation in the *NF1* gene that encodes a neurofibromin protein. This mutation leads to the production of a mutated form of the protein. The *NF1* gene encodes a large protein called neurofibromin that consists of 2818 amino acids and has a GTPase activating protein (GAP)-related domain that interacts with p21 and inhibits RAS signaling [7,8,9]. Neurofibromin also contains a Sec14-pleckstrin homology (PH) module, which consists of a Sec14-like protein that is identified in yeast as a lipid binding domain, and a pleckstrin-like homology domain that binds phosphatidylinositol. Although this module can bind to phospholipids, its exact function is unclear [10]. *NF1* is ubiquitously expressed and is most highly expressed in neuronal cells. It acts as a tumor suppressor gene that prevents the uncontrolled proliferation of cells via negative regulation of RAS growth signaling. Studies have also shown that mice lacking the *Nf1* gene died during embryonic development because of heart developmental defects. These results confirmed that the *NF1* gene plays a critical role in embryonic survival [11].

Multiple mutations in the *NF1* gene have been identified as the cause of NF1 disease. The genotype-phenotype correlation has been documented, and studies have revealed that some genetic variants are associated with severe symptoms [12,13,14]. Multiple systems, including the nervous, integumentary, ophthalmic, pulmonary, musculoskeletal, and cardiovascular systems, are affected by NF1. NF1 is also associated with an increased risk of benign and malignant tumors, such as neurofibroma, malignant nerve sheath, glioma, glioblastoma, duodenal carcinoids, breast cancer, gastrointestinal stromal, hematological, pheochromocytoma, and rhabdomyosarcoma [15].

Neurofibromatosis animal models have helped to elucidate the underlying pathogenesis of this disease and have aided in the development of various treatments that counteract pathogenic pathways and improve the management of the disease.

This review summarizes the latest breakthroughs in drug development for NF1-associated tumors and briefly discusses the development of NF1 genetic models.

## 2. Benign Nerve Sheath Tumors (Neurofibroma)

Neurofibroma is a hallmark of NF1 that is divided into the following four subtypes: cutaneous, plexiform, subcutaneous, and spinal neurofibroma. The most common forms, plexiform neurofibroma (PN) and cutaneous neurofibroma [15], are discussed in this section.

The plexiform subtype arises from multiple fascicles along the nerve. This neurofibroma subtype can be seen anywhere in the body [15]. Although it is a benign condition, it is associated with significant morbidity and has the potential to develop into a life-threatening condition [16]. Additionally, this neurofibroma form can develop into a malignant nerve sheath tumor, which is an aggressive soft tissue tumor that has a lifetime risk of 8–13% [17]. Conversely, the risk of cutaneous neurofibroma malignancy is null; however, cutaneous neurofibroma can cause itching, pain, and disfigurement that all significantly impact the quality of life [18]. Although surgery is the main treatment option for PN, it is associated with a high recurrence rate due to incomplete surgical resection. Procedural treatments are usually performed for cutaneous neurofibroma.

Dysregulation of signaling pathways was found to play a role in neurofibroma growth, and several drugs that counteract this aberrant signaling have been developed.

### 2.1. Targeting RAS Signaling

The NF1 protein is a negative regulator of RAS signaling; therefore, the loss of NF1 protein leads to the overactivation of the RAS signaling pathway and the development of tumors [19]. Hiatt and colleagues showed that *Nf1* deficient murine cells were hyperproliferative and more sensitive to growth signaling compared to controls. Expressing the NF1- GAP-related domain (GRD) in these cells halted their aberrant growth and abolished their hypersensitivity to growth factors. This process was partially mediated via the inhibition of RAS signaling because it was observed following the correction of extracellular signal-regulated kinase (ERK) and AKT activity [20]. Pharmacological interference with RAS signaling was found to hamper the growth of NF1-nerve tumors in preclinical studies. Additionally, it was reported that inhibition of the RAS-downstream effector mitogen-activated protein kinase (MEK) kinase in the *Nf1^flox/flox^; DhhCre* mouse model of NF1 hampered the growth of neurofibroma in 80% of mice. Importantly, this effect was observed at low doses that could be used to reduce the toxic effect of the MEK inhibitor while preserving its efficacy [21].

In clinical studies, MEK inhibitors were a successful therapeutic approach for neurofibroma treatment. Results from a phase I clinical trial revealed that the MEK inhibitor, selumetinib, induced a response rate (defined as at least 20% reduction in PN volume from baseline) in 71% of pediatric patients with an acceptable toxicity profile and no disease progression. Other than its unprecedented effect on tumor volume, selumetinib treatment was associated with improvements in motor function, alleviation of pain, and reduction of disfigurement [22]. These encouraging results paved the way to phase II clinical trials. Results from phase II clinical trials revealed that selumetinib induced a partial response rate in 70% of patients with inoperable PN, and 56% of the patients had a durable response. Moreover, at least one PN-related complication was improved in 68% of patients [23]. Selumetinib has recently been granted FDA approval for symptomatic, inoperable PN in pediatric patients (i.e., patients who are two years of age and older), making it the approved first targeted therapy for PN. To reduce the toxicity associated with selumetinib, a clinical trial focusing on the intermittent dose of selumetinib in children with inoperable PN is ongoing (NCT03326388) [24]. In adult patients, a phase II study of selumetinib is also ongoing (NCT02407405) [25]. In addition to PN treatment, selumetinib is currently being tested in a pilot phase II clinical trial for patients with cutaneous neurofibroma (NCT02839720) [26].

Additionally, MEK inhibitors may be used as a modality to enable surgery. For example, trametinib was administered to a girl with a massive PN in the neck region that was associated with a significant cervical spinal deformity for six months. Treatment with trametinib led to a 22% reduction in tumor volume, and the case became eligible for surgery [27]. Trametinib (NCT03363217) [28] and other MEK inhibitors, such as biminitib (NCT03231306) [29] and mirdametinib (NCT03962543) [30], are currently being testing in phase II clinical trials.

Although MEK inhibitors delayed the formation of neurofibroma and increased the response rate as compared with other drugs that were tested, they did not prevent the development of neurofibroma [31].

Another approach to counteract RAS signaling is by interfering with RAS farnesylation, which is a fundamental process for biologically active RAS. This process is mediated by an enzyme called farnesyltransferase (FTase) [32]. The FTase inhibitor was shown to inhibit the growth of NF1 malignant peripheral sheath tumor cell lines [33]. However, the FTase inhibitor, tipifarnib, did not significantly prolong the time to progression (TTP) in active PN cases. Nevertheless, it improved the quality of life according to scores on the Impact of Pediatric Illness (IPI) scale. These significant improvements were specifically observed in the emotional domain, and there was also a trend toward cognitive improvement [34].

### 2.2. Targeting Mammalian Target of Rapamycin (mTOR)

The mTOR signaling pathway plays a fundamental role in various physiological processes. Dysregulation of this pathway has been implicated in many pathological conditions, such as cancer [35]. The mTOR signaling pathway was dysregulated in *Nf1^−/−^* mouse embryonic fibroblast cells, and the growth of these cells was apparently inhibited by rapamycin (known as sirolimus in the clinic), which is an mTOR inhibitor [36]. Although preclinical studies have suggested that mTOR inhibitors play a potential role in inhibiting the growth of NF1-related nerve tumors [37,38,39], the results were not encouraging in clinical studies. In phase II studies, rapamycin, which is an FDA approved mTOR inhibitor, showed moderate prolongation of TTP (4 months) but did not decrease tumor size. Studies have also revealed that the response to rapamycin varies between patients. For example, some patients showed a 30% doubling in TTP as compared with 7.5% of historical controls [40]. Everolimus, which is another mTOR inhibitor, was tested in a small group of patients with cutaneous neurofibroma and showed a significant response, suggesting that this treatment may provide possible benefits for patients with cutaneous neurofibroma [41].

### 2.3. Targeting Microenvironments

Microenvironments play a pivotal role in tumor development and progression [42]. Inflammation is a hallmark of cancer and has been recognized as a main factor in tumor development [43]. It is also hypothesized that inflammation mediates neurofibroma initiation. PN is a mixture of various cell types, including neuronal cells, fibroblasts, immune cells, endothelial cells, and pericytes. The importance of heterotypic interaction in PN is well documented. Mast cells are recruited to the tumor site and are activated by ligands that are produced by tumor cells to release growth factors that affect tumor cells and the microenvironment. Fibroblasts proliferate and deposit extracellular matrix in response to the factors that are released by mast cells, and this leads to an increased tumor mass. Moreover, angiogenic factors released by various inflammatory cells and tumor cells enhance angiogenesis and facilitate tumor growth [44,45]. Early clinical studies revealed that the mast cell stabilizer, ketotifen, induced beneficial effects in patients with cutaneous neurofibroma [46,47,48]. In various mouse models, mast cells were pivotal for neurofibroma development and growth. Yang and colleagues showed that heterozygous mast cells were required for neurofibroma development via the c-KIT axis. They also found that treatment with imatinib mesylate, which inhibits c-KIT signaling and counteracts mast cells, mediated pro-tumorigenic effects and ultimately reduced tumor size. This FDA approved drug was used off-label to treat a child with a life-threatening PN. The result was fascinating, and the patient experienced a significant improvement in her condition [49]. Subsequently, a phase II clinical trial using imatinib mesylate revealed that there was a ≥20% reduction in tumor size in 26% of PN patients who received the treatment. Moreover, 30% of subjects reported improvements in quality of life. At the individual level, there was heterogeneity in the response rate, but some patients showed marked responses that reached 38% [50]. Other drugs that inhibit c-KIT, such as nilotinib, are more potent and less toxic than imatinib [51]. A phase I nilotinib clinical trial was conducted (NCT01275586) but the results have not been published [52].

Other inflammatory cells also play a role in NF1 pathogenesis. Macrophages play a significant role in tumor development [53] and are a predominant cell type in PN; they constitute 20–40% of the tumor cells. Decreasing macrophages by signal transduction and activation of transcription 3 (STAT3) inhibitor halted neurofibroma growth in a neurofibroma mouse model (*Nf1 ^flox/flox^ ; DhhCre*) [54]. Animal studies also showed that depletion of macrophages by PLX3397, which is a selective colony-stimulating factor-1 receptor (c-Fms) and c-KIT inhibitor that inhibits macrophage and mast cells recruitment, decreased the growth of neurofibroma. However, the role of macrophages differs in later stages of tumors as compared with early stages. For example, treatment with pexidartinib (PLX3397) worsened the neurofibroma phenotype when administered at an early stage in the disease [55]. PLX3397 is currently investigated in clinical trials for PN (NCT02390752) [56]. Interestingly, imatinib was also shown to influence macrophages [57]. Additionally, since macrophages are recruited via CXCL10-CXCR3, which are expressed in T cells and dendritic cells, the deletion of CXCR3 may prevent neurofibroma formation. However, studies revealed that anti-CXCR3 did not reduce the tumor size of the established tumor [58].

An alternative approach to inflammatory cell-targeting is evoking an immune response. Interferon alpha is a cytokine that belongs to an intronless family of genes called type 1 interferon. Type 1 interferons play a fundamental role in cell defense against infections and have been implicated in various inflammatory pathological conditions [59,60]. The role of type 1 interferons in cancer is complex and context dependent because it can be either anti- or pro-tumorigenic [61]. Nevertheless, interferon alpha 2b is approved for the treatment of some types of cancers [62]. In a phase II clinical trial, the TTP in patients with active PN who were treated with pegylated interferon was prolonged as compared with that in historical placebo controls (29.4 and 11.8 months, respectively) [63]. Toll-like receptors (TLRs) are a family of proteins that is involved in innate immunity. They sense microbes that are derived or from self-damaged cells and activate downstream pathways that promote host defense mechanisms. TLR7 is an intracellular TLR protein that mainly senses single stranded RNA and is predominantly expressed in plasmacytoid DCs (pDCs). Stimulation of this receptor enhances innate immunity, and it has been used in some skin conditions, such as basal cell carcinoma [64,65]. Imiquimod, a TLR7 agonist, was tested and found to have only minimal effects on cutaneous neurofibroma [66].

Angiogenesis is another main contributor to tumor development, and drugs targeting angiogenesis are used for the treatment of many cancers [67]. Many drugs that were tested in clinical trials, such as MEK1 and mTOR inhibitors, may be useful for the treatment of neurofibroma because they affect tumor vasculature [68]. However, anti-angiogenic multikinase inhibitors, such as sorafenib and sunitinib, failed in clinical trials because of safety concerns [69,70]. In a phase II clinical trial of progressive or non-operable PN, cabozantinib, which is a tyrosine kinase inhibitor of c-KIT, hepatocyte growth factor receptor (c-MET), vascular endothelial growth factor (VEGF), and AXL achieved a partial response in 42% of patients as evidenced by ≥20% reduction in volume on MRI with no progression noted in any of the treated patients [71]. Additionally, ongoing clinical trials are being conducted in pediatric patients (NCT02101736) [72]. The effect of the anti-angiogenic drug, ranibizumab, was minimal in cutaneous neurofibroma [66]. Other clinical trials that have assessed other microenvironmental factors showed no effect on neurofibroma. For example, phase I and II trials of pirfenidone, an antifibrotic agent, failed to show any effect on tumor volume, TTP, or quality of life [73].

### 2.4. Runt-Related Transcription Factor (RUNX) Inhibition

The runt-related transcription factor (RUNX) family consists of RUNX1, RUNX2, and RUNX3 transcription factors. These transcription factors play a fundamental role in normal development. RUNX1 is essential for hematopoiesis, RUNX2 is important for skeletal development, and RUNX3 plays a fundamental role in neurogenesis. These transcription factors also play a role in the development of other organs. RUNX factors have been implicated in various types of cancer [74]. By comparing gene expression between tumor-initiating cells and the remainder of tumor cells in neurofibroma, Li and colleagues found that the *RUNX1* gene was highly overexpressed in human tumor initiating cells. Specifically, the expression of *RUNX1* in tumor initiating cells was seven times higher than that in the remaining tumor cells. Notably, it was overexpressed in neurofibroma lesions with minimal expression in wild type cells. Inhibition of *Runx1* delayed the formation of neurofibroma in mouse models [75]. Importantly, *Runx1* inhibition resulted in compensatory *Runx3* overexpression. Dual deletion of *Runx1/3* genes prolonged the survival of a neurofibroma mouse model and reduced the number of neurofibroma lesions. Ro5-3335, a RUNX1/CBFβ inhibitor, induced tumor cell death and led to tumor volume shrinkage [76].

## 3. Malignant Nerve Sheath Tumor

**Figure 1:**
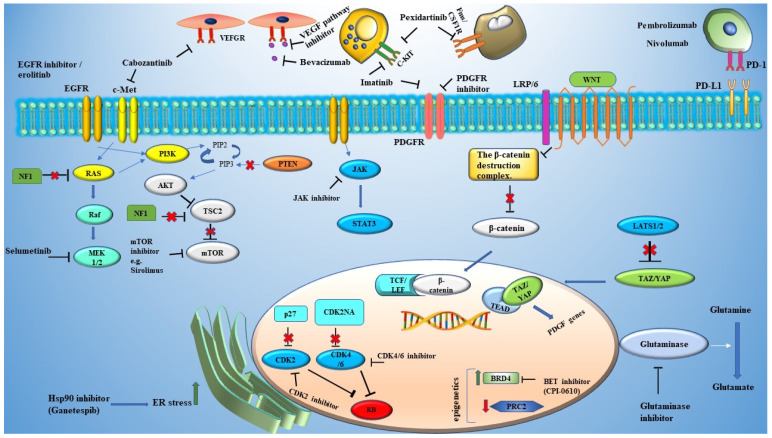
Targetable pathways in NF1. A plethora of signaling pathways has been implicated in MPNST growth and progression. These pathways include growth signaling pathways, tumor suppressors, pathways that are involved in metabolism and development, and pathways that are involved in angiogenesis and immune responses. Pathways and their targeted drugs are represented schematically in this figure. Loss of NF1 protein leads to the activation of RAS/RAF/MEK and TSC2/mTOR signaling pathways, which support cancer cell proliferation. Dysregulation of cell cycle checkpoints, such as CDK2NA and p27, led to abnormal levels of cell cycle kinase CDK4/6 and CDK2, which support cancer cell growth and proliferation. Growth receptor aberrations also contribute to MPNST pathogenesis. Examples of the aberrant receptor signaling that can be targeted are EGFR, c-Met, and PDGFR. Some signaling pathways that are involved in cell development and stemness have been implicated in MPNST development. These are represented by the Wnt and Hippo pathways. Targeting tumor microenvironments is another strategy to treat MPNSTs. Drugs that target the tumor microenvironment include bevacizumab, which targets pro-angiogenic VEGF pathways, and imatinib and pexidatrininb which target mast cells and macrophages, respectively, and play pro-tumorigenic role. Immune checkpoint inhibitors are other examples of drugs in this category. Other targets that are represented are involved in metabolism (glutaminase), epigenetic (BRD4), and ER stress (Hsp90).

Malignant peripheral nerve sheath tumors (MPNSTs) are aggressive soft tissue tumors. Approximately 50% of MPNST cases are developed in patients with NF1. The 5-year survival rate of patients with MPNST is 44%, and the 5-year progression-free survival is 46%. Treatment options are limited and include surgery, which is currently the main intervention, radiology, and chemotherapy [77]. Therefore, other treatment modalities are necessary. Since many of these tumors arise in the context of an *NF1* gene mutation, targeting pathways that are regulated by NF1 protein may offer prevention/treatment options. Additionally, these malignancies harbor other mutations, such as p53, cyclin—dependent kinase inhibitor 2A (CDKN2A), and polycomb repressive complex 2 (PRC2) components that may be targeted pharmacologically [78,79,80]. Many tyrosine kinase receptors (TKR) are overexpressed in MPNSTs. Therefore, inhibition of TKRs signaling may be another viable option for MPNST treatment [81]. Targetable dysregulated pathways are summarized in Figure 1.

Epidermal growth factor receptor (EGFR) expression was also shown to be elevated in MPNSTs [82]. A preclinical study on erlotinib, which inhibits EGFR, showed inhibition of the growth of these cells [83]. However, the targeted treatment effect in clinical trials was not achieved [84].

Preclinical studies showed that mTOR pathway inhibitors may be promising treatments for MPNSTs [85]. An approach to MPNSTs management involves a dual targeting strategy to inhibit mitogen-activated protein kinase (MAPK) and mTOR and synergize the growth inhibition of cancer cells to achieve a longer response [86]. An ongoing phase II clinical trial is currently assessing the combination of selumetinib (MEK inhibitor) and rapamycin (mTOR inhibitor) in MPNSTs [87]. A combination of everolimus (mTOR inhibitor) and bevacizumab (anti-angiogenic) was also used in a clinical trial, but it did not achieve the targeted treatment benefits [88]. Other combinations were used and are discussed in the following section.

### 3.1. Developmental Genes in MPNSTs

Many pathways that play a role in development have been implicated in tumorigenesis. The Hippo pathway is a conserved cell signaling pathway that is crucial for embryonic development and controlling the size of organs. Defects in these pathways also contribute to many diseases. In vertebrae, the transcriptional co-activator with a PDZ-binding domain (TAZ)/Yes-associated protein (YAP) is the main effector pathway of the Hippo pathway that has been implicated in many physiological and pathological conditions. TEA domain family member (TEAD) transcription factors are essential for many TAZ/YAP processes. TAZ/YAP are negatively regulated by large tumor suppressor 1 and 2 (LATS1/2) protein [89]. In cancer, Hippo pathway dysregulation affects biological pathways that are central to tumorigenesis [90]. MPNSTs have aberrant TAZ/YAP signaling activation and loss of *LATS1/2* expression. Using *Lats1/2* ablation as an NF1 model was associated with the development of highly aggressive MPNSTs with apparent TAZ/ZAP hyperactivity. Inhibition of TAZ/YAP impeded tumor growth in *Lats1/2*-deficient mice.TAZ/YAP-TEAD was shown to activate many oncogenic genes. Notably, robust activation of PDGF signaling genes was noted. Dual inhibition of TAZ/YAP and PDGFR has a synergistic effect on those tumors [91].

Another important signaling pathway is the Wnt signaling pathway. This pathway is an essential cell signaling that has been implicated in many physiological and pathological conditions. Dysregulation of the Wnt signaling pathway contributes to the development and progression of many cancers [92]. It was also reported that the activation of Wnt/B catenin signaling by the downregulation of Wnt negative regulators or by the activation of their ligands affected MPNST cell behavior. Cell viability in MPNSTs was significantly decreased following treatment with a Wnt signaling inhibitor as compared with that following treatment with a control. Furthermore, the Wnt signaling inhibitor had a synergistic effect with an mTOR inhibitor to induce cell death [93].

### 3.2. Endoplasmic Reticulum (ER) Stress in MPNSTs

Endoplasmic reticulum (ER) stress is a cellular phenomenon that occurs as a result of the accumulation of misfolded proteins at the ER lumen. ER stress is caused by many stressful conditions that alter the balance between the levels of proteins that require folding and the protein folding capacity of the ER. To avoid the detrimental effects of ER stress, cells are armed with an adaptive mechanism called the unfolded protein response (URP). Tumors are subjected to various ER stress-induced conditions both intrinsically and extrinsically more than normal cells and URP in cancer cells has been linked to hallmarks of cancer [94]. Thomas De Raedt and colleagues found that the ER stress of MPNSTs was higher than that of normal cells [95]. In an in vitro study, they also found that treatment with Hsp90 inhibitor; which is an ER stress-inducing drug, induced cell death in malignant cells; however, normal cells were spared. Although in vivo studies of Hsp90 inhibitors alone were not sufficient to regress tumors, tumor regression was observed when Hsp90 inhibitors were combined with rapamycin. The combination has a damaging effect on ER and mitochondria and triggers reactive oxygen species (ROS) production while suppressing antioxidant systems [95]. Ganetespib (Hsp90 inhibitor) treatment was tested in combination with rapamycin in patients with refractory sarcoma and MPNST. The combination was well-tolerated but there was no observed response [96]. This possibly was due to the characteristics of study participants who were heavily pretreated and had refractory tumors [96].

### 3.3. Targeting Cancer Metabolism

Cancer cells have an aberrant metabolism that evolved to help cancer growth, and many metabolic pathways are involved in tumorigenesis [97]. It was found that glutamine is essential for NF1—associated MPNST cell line proliferation [98]. Moreover, cell proliferation and tumor volume were reduced in xenograft models that were treated with glutaminase inhibitors as compared with those in controls [98]. A phase II study (NCT03872427) that was initiated to investigate the role of CB-839 HCl, a glutaminase inhibitor, in cancer cases with specific mutations, including NF1-mutation positive MPNSTs, is still ongoing [99].

### 3.4. Epigenetic and MPNSTs

Epigenetic components have emerged as therapeutic targets for various cancers [100]. Among these compounds are bromodomain and extra-terminal motif (BET) proteins. Multiple BET inhibitors have been developed and many are being tested in clinical trials [101]. In NF1, it was noted that levels of BRD4, which is a member of BET proteins, were elevated in mouse models of NF1 and a pro-tumorigenic effect was observed. Further, pharmacological inhibition using JQ1, a pan BET inhibitor, led to 50% near complete shrinkage of tumor volume [102]. Another study showed that BRD4 levels affected the sensitivity to BET inhibitors and that BRD4 inhibitors rendered cells more sensitive to BET inhibitors [103]. PRC2 loss was shown to be mutated in 70% of NF1- MPNSTs [104]. Interestingly PRC2 function loss rendered the MPNSTs sensitive to BET, DNA methyltransferase, and histone deacetylase (HDAC) inhibitors [105,106]. When combined with the MEK inhibitor, the BET inhibitor exerted a profound effect on MPNSTs growth [105]. Additionally, data from other studies showed that there was a synergic effect between BET inhibitors with immunotherapy and HDAC inhibitors [101]. Although a phase II clinical trial for the BET inhibitor, CPI-0610, was initiated in patients with MPNSTs, it was withdrawn because of lack of enrollment (NCT02986919) [106].

### 3.5. Harnessing the Immune System

Immunotherapy in cancer has gained immense interest in the last 10 years because it has shown unprecedented success in the treatment of many cancer types [107]. In NF1, ongoing clinical trials are being conducted to test the efficacy of pembrolizumab in MPNSTs (NCT02691026) [108] and the combination of nivolumab and ipilimumab in rare tumors, including MPNST (NCT02834013) [109].

Another class of drugs that was investigated is oncolytic viruses. Oncolytic measles virus (MV) expressing sodium iodine symporter gene (MV-NIS) induced cell death in multiple MPNST cell lines with minimal effect on normal cells [110]. In xenograft models, mice who were treated with MV-NIS had a significantly prolonged survival rate with differences in susceptibility between MPNST lines that were used [110]. This viral treatment is now under clinical assessment in a phase I study (NCT02700230) [111]. The anti-tumor effect was also observed using the oncolytic herpes simplex virus (oHSV) G47Δ. Studies showed that there was a 30% prolongation in survival with histological evidence of massive necrosis in both immunodeficient and immunocompetent mice [112]. A more prominent anti-tumor effect was observed when these viruses were armed with IL-12 [112]. A phase I trial was initiated for HSV1716 in variable, non-CNS tumor types, including MPNSTs (NCT00931931) [113].

## 4. Glioma

The risk for glioma in NF1 patients is high. Low grade gliomas are more common in pediatric groups whereas high grade gliomas are more common in adults [114]. Optic gliomas are the most commonly encountered brain tumors in children and affect 15–20% of patients with NF1 [115]. The prognosis of optic gliomas is good, and only one-third of patients require intervention; however, this disease can lead to vision loss and pituitary complications in some cases. The current first line treatment for progressive optic glioma is chemotherapy (carboplatin/vincristine) [115].

Overactivation of mTOR and MEK is also predominant in gliomas. Inhibition of these pathways halts the growth of optic gliomas and ameliorates retinal abnormalities [116,117]. Selumetinib treatment achieved partial progress in 40% and visual acuity improvement in 20% of NF1 low grade glioma in a phase II clinical trial [118]. Comparisons between selumetinib and standard treatment, carboplatin/vincristine, are ongoing in a phase III trial (NCT03871257) [119]. Everolimus (an mTOR inhibitor) has also been recently tested in a phase II clinical trial for treatment of low grade gliomas in patients with NF1 (NCT01158651); however, the results of this clinical trial have not been published yet [120].

Angiogenesis is another target for the treatment of glioma. In animal models, angiogenesis precedes tumor formation, and vessel density was associated with a poor prognosis in humans [121,122]. Several reports showed that the anti-angiogenic drug, bevacizumab, improved the clinical outcome of optic gliomas [123,124,125,126,127]. Ongoing clinical trials for glioma are summarized in Table 1. The immune system is another important factor because it plays a pivotal role in glioma development. Preclinical studies demonstrated that glioma growth is dependent on factors from microglial cells, and inhibition of microglial function halts tumor growth [128]. This process is thought to be mediated by chemokine ligand 5 (CCL-5) secretion. The expression of CCL-5 increases ten times in *Nf1^+/−^* microglia within optic glioma compared to *Nf1^+/−^* microglia present in normal optic nerve [129]. Anti-CCL-5 inhibited the proliferation of optic gliomas by 9.8-fold and ameliorated retinal damage [129]. Yuan Pan and colleagues studied gene expression in four different *Nf1*-optic glioma models and found a 25-gene expression signature that is shared between all glioma models compared to normal optic nerve [130]. Notably, minocycline treatment, which blocks microglial function, neutralized the expression changes in glioma models [130]. T cells also contribute to the pathogenesis of gliomas. Athymic mice exhibited reduced CCL-5 expression in the brain, abnormalities in microglial cells, and inhibition of tumor formation [131]. T cell infiltration is mediated by chemokines that are released by cancer stem cells. T cells enhance secretion of CCL-5 by microglial cells, which in turn affect optic glioma stem cells and provide a cycle of interactions between these elements [132]. However, there is evidence for immune surveillance in glioma. For example, transcriptome analysis indicated heavy immune cell infiltration and the presence of activated anti-tumor T cells loaded with glioma-neoantigen [133]. This presents an opportunity to tailor immunotherapy for a subset of patients.

## 5. Non-Coding RNA in NF1 Tumors

Protein coding genes are a major focus in cancer research. Although the roles of protein coding genes are well established and undeniable, they still do not reflect cancer in its entirety. Ninety percent of RNAs in cells are non-coding RNAs. These non-coding RNAs were previously classified as junk, but they are now recognized as having a wide biological function in both physiological and pathological conditions. In cancer, non-coding RNAs play a significant role in tumor development and can be utilized as a biomarker for many types of cancer. RNA-based therapeutics have also been investigated in clinical settings [144].

The expression of many non-coding RNAs was found to be dysregulated in NF1-related tumors. Julin and colleagues compared the expression of 377 miRNAs in benign and malignant nerve sheath tumors [145]. They found that miR-486-3p, which targets the tumor suppressor PTEN, and miR181a, which targets the tumor suppressor ATM, were aberrantly overexpressed in PN as compared with that in dermal neurofibroma. However, a set of miRNAs that negatively regulate the RAS-MAPK pathway were also overexpressed in PN. Comparing NF1- MPNSTs with PN, they found 113 miRNAs that differed in expression between the two groups. Dysregulated miRNAs affect known pathological pathways in cancer such as PTEN, RAS-MAPK, epithelial-mesenchymal transition, and cell cycle progression [145]. Azadeh and colleagues also studied the miRNA expression profile of PN, NF1- MPNSTs, and non-NF1- MPNSTs [146]. They found that the miRNA profile helped to distinguish between NF1- PN, and NF1- MPNST and between NF-1 MPNST and the sporadic form of MPNST. They functionally validated six miRNAs among the top 15 differentially expressed miRNAs, including miR-889 which was not reported before. They showed that miR-889 along with miR-135b promote Wnt/B-catenin signaling and contribute to the invasive and migratory capabilities of MPNST cells [146]. The Let7 family is also part of a well-known family that plays a role in cancer. Let7b was downregulated in MPNSTs where it acts as a tumor suppressor that inhibits cell proliferation and migration [147].

MiR-612 is another tumor suppressor miRNA that was studied. Transfection of miR-612 in MPNST cells reduced tumor proliferative and migratory capabilities [148]. Moreover, the xenograft model derived from NF1-MPNST overexpressing miR-612 showed decreased tumor volume and proliferative markers. The anti-tumor effect is believed to be mediated via controlling the level of Fas apoptotic inhibitory molecule 2 (FAIM2) [148]. Additionally, dysregulated miRNAs are regulated by well-known proteins. For example, miR10-b is regulated by TWIST, and miR-34a is regulated by p53 [149,150]. miR-10b acts as oncogene in NF1-MPNSTs, and levels of miR-10b were elevated in NF1-MPNSTs as compared with those in non-NF1-MPNSTs [149]. Many well characterized proteins that are targeted by miR-10b may contribute to its tumor promoting function. miR-10b negatively regulates NF1 protein expression and augments the RAS signaling pathway. However, an NF1 protein-independent mechanism is also possible. miR-10b also promoted the expression of Ras homolog gene family member C (RhoC), which is a metastasis-promoting gene. Inhibition of miR-10b alleviated the malignant behavior of MPNST cells [149]. miR-34a is a tumor suppressor miRNA that was downregulated in NF1-MPNSTs because of p53 inactivation. Notably, it is possible that mi-34a loss significantly contributes to MPNST development [150]. miR-34a ectopic expression induces apoptotic cells death in MPNST cells, and miR-34a transfection showed similar experimental results with p53 ectopic expression [150]. Although RNA-based treatment is still in its infancy, studies that have highlighted the importance and role of non-coding RNA encourage their utilization for the treatment of NF1 tumors.

## 6. “Hit the Bullseye”

Gene therapy has progressed rapidly in the last decade, and advancements have been made in many aspects, including gene modification methods, gene delivery systems, safety, etc. [151]. Gene therapy has been investigated as a way to cure various inherited diseases and tumors. Some gene-based therapies have been approved and are available for treatment [151]. Given that the NF1 genetic mutation is essential for NF1-related diseases, targeting the gene defect may lead to a cure.

NF1-GRD-based adeno-associated virus (AAV) vectors restore the function of normal genes and suppress the growth of MPNSTs [152]. Although gene therapy is promising, there are many challenges that need to be further studied. In NF-1, gene therapy is still in its infancy, and ongoing efforts are being made to develop drugs that can be used in humans to successfully correct the original defect [153].

## 7. Genetically Engineered Models for NF1

Animal models have deepened our understanding of NF1 related tumors pathogenesis. Murine models are widely used, and various generations have been developed to recapitulate many of the pathological features seen in human tumors.

Classical mouse models carrying heterozygous *Nf1* mutation were developed by introducing disruptive neomycin (neo) cassettes in one allele [44,154]. These models may develop some features seen in human NF1 disease and increase risks for some tumors such as pheochromocytoma and leukemia. Unlike humans, these models do not develop the hallmark features of NF1 such as PN and nervous system tumors which makes them not useful for NF1 related tumor studies [44,154]. However, these models provided evidence of the requirement of biallelic inactivation of *NF1* to develop tumors. Unfortunately, the embryonic lethality of *Nf1^−/−^* mutation makes it impossible to use mice carrying such mutations as tumor models.

To overcome this, chimeric *Nf1^−/−^* mice and conditional knockout models were then developed. Chimeric *Nf1^−/−^* mice harbor many of the hallmark features of NF1 disease including neurofibroma, asserting the need for biallelic inactivation in neurofibroma development [44,154]. Conditional knockout mice models give more insight about the cell of origin, role of microenvironment, and time needed for harboring mutations. For such models, *Cre-lox* site-specific recombination technology was traditionally used [44,154]. For PN, the *Nf1^flox/flox^; Krox20Cre* mouse model was developed to achieve biallelic inactivation of *Nf1* gene in Schwann cells and their precursors while having wild type *Nf1* in other somatic cells. However, these cells did not develop tumors. A modified model called *Nf1^flox/−^*; *Krox20Cre* was then used to achieve biallelic inactivation in Schwann cells and heterozygous *Nf1* in other cells. These models developed neurofibroma suggesting the role of both biallelic inactivation in tumor cells and heterozygosity in stromal cells. Another model is the *Nf1^flox/flox^*; *DhhCre* model in which a widespread embryonic biallelic inactivation is achieved in glial precursor cells. Notably, in this model, there is no need for haploinsufficient stromal cells. *Nf1^flox/−^; Krox20Cre* model is better at mimicking the scenario in humans because stromal cells in NF1 related tumors are haploinsufficient, while *Nf1^flox/flox^; DhhCre* can represent sporadic tumors that harbor *NF1* mutations. Models for optic glioma tumors demonstrate the same concept. Biallelic activation for *Nf1* in astrocytes depends on haploinsufficient stromal cells to develop a tumor, while biallelic activation in earlier progenitor cells bypasses such dependency suggesting that timing of the mutation determines the NF1 required dosage to develop tumors [44,154].

*NF1* mutation alone is not sufficient to develop MPNSTs. To do so, concomitant loss of function of *p53*, *Pten* or *Ink4a,* or *Egfr* amplification along with *Nf1* mutation is introduced in NF1-related MPNST models [44,154,155]. To better mimic the tumor pathology in humans, scientists developed a model in which *Nf1* and *p53* loss arise sequentially rather than concomitantly [155]. Recently, CRISPR/Cas9 has also been utilized in NF1-related tumor models [156]. Other commonly used models to investigate MPNSTs and drug development are MPNST-xenograft models [21,98,157].

All genetically engineered models can be also modified by crossing them with mice that have specific genetic defects to assess the contribution of specific genes on the NF1- related tumors in tumor cells or their microenvironments [58,76,91]. Apart from commonly used mice models, scientists also developed a minipig model for NF1 which may overcome some limitations in mouse models and better represent disease features in humans [158,159].

## 8. Conclusions

A deep understanding of node signaling pathways that are involved in the development of NF1 has led to the development of targeted therapies that have been successful in clinical trials and approved by the FDA. The tumor microenvironment is a fundamental player in tumorigenesis, and its target has been used to control tumor growth. Targeting microenvironments has been used for cancer treatment, and it is a promising approach for the treatment of NF1-related tumors. Immunotherapeutic drugs are under investigation in clinical trials for MPNSTs, and theoretical evidence supports their use in NF1 glioma. Other studies have also shed the light on more targeted pathways in metabolism, ER pathways, and epigenetics. Notably, non-coding RNA has emerged as a contributor to NF1; however, more studies are required to investigate the effect of non-coding RNA on NF1-related tumors and to determine the potential for RNA-based therapies to be used as a possible treatment modality. Identifying different druggable signaling aberrations may pave the way to personalized medicine for MPNSTs in the future. Gene therapy for NF1 is under investigation and is the potential cure for the disease. However, gene therapy may not be a viable choice for MPNSTs because the tumors harbor multiple mutations.

## Figures and Tables

**Table 1:** Ongoing clinical trial for drugs in NF1 glioma.

Drug	Patients	Mechanism of Action	Clinical Trial
Selumetinib	Young patients with recurrent or refractory low-grade glioma	MEK inhibitor	NCT01089101 Phase I/II [134]
Lenalidomide	Low dose or high dose in younger patients with recurrent, refractory, or progressive pilocytic astrocytoma of optic pathway glioma	Anti-angiogenesis, tumor apoptosis, and immunomodulatory effect [135]	NCT01553149 Phase II [136]
Pegylated interferon alfa-2b (PEG-Intron)	Children with juvenile pilocytic astrocytoma and optic pathway gliomas	Activate (JAK/STAT) pathway	NCT02343224 Phase II [137]
Trametinib	Pediatric neuro-oncology patients with refractory tumors and activation of the MAPK/ERK pathway	MEK inhibitor	NCT03363217 Phase I/II [28]
MEK162	Children with RAS/RAF pathway activated tumors	Selective inhibitor of MEK	NCT02285439 Phase I/II [138]
Vinblastine +/− Bevacizumab	Children with unresectable or progressive low-grade glioma (LGG)	Alkaloid chemotherapy and antiangiogenetic	NCT02840409 Phase II [139]
Pomalidomide	Younger patients with recurrent, progressive, or refractory central nervous system tumors	Anti-proliferative and immunomodulator	NCT02415153 Phase I [140]
Dabrafenib, Trametinib and Hydroxychloroquine	Patients with recurrent LGG or HGG with a BRAF aberration	BRAF inhibitor, MEK inhibitor, and autophagy inhibitor	NCT04201457 Phase I/II [108]
Poly-ICLC	Low-grade gliomas in pediatric patients with NF1	Immune stimulant, Toll-like receptor-3 agonist [141]	NCT04544007 Phase II [142]
Dabrafenib and trametinib	Patients with BRAF V600 mutation positive low-grade glioma or relapsed or refractory high-grade glioma.	BRAF inhibitor and MEK inhibitor	NCT02684058 [143]
